# Alcohol use in the military: associations with health and wellbeing

**DOI:** 10.1186/s13011-015-0023-4

**Published:** 2015-07-28

**Authors:** Michael Waller, Annabel C. L. McGuire, Annette J. Dobson

**Affiliations:** The University of Queensland, Centre for Australian Military and Veterans Health, School of Public Health, Herston Road, Herston, 4006 Australia; The University of Queensland, School of Public Health, Herston Road, Herston, 4006 Australia

**Keywords:** Alcohol, Military, Health, Drinking, General population

## Abstract

**Background:**

This study assessed the extent to which alcohol consumption in a military group differed from the general population, and how alcohol affected the military group’s health and social functioning.

**Methods:**

A cross sectional survey of military personnel (*n* = 5311) collected self-reported data on alcohol use (AUDIT scale) and general health, role limitations because of physical health problems (role physical), and social functioning scores (SF36 subscales). Logistic regression was used to compare drinking behaviours between the military sample and a general population sample, using the categories risky drinkers (>2 units per day), low risk drinkers (≤2 standard drinks per day) and abstainers. Groups in the military sample with the highest levels of alcohol misuse (harmful drinking AUDIT ≥ 16, alcohol dependence AUDIT ≥ 20, and binge drinking) were also identified. Linear regression models were then used to assess the association between alcohol misuse and SF36 scores.

**Results:**

There were fewer risky drinkers in the military sample than in the general population sample. There were also fewer abstainers, but more people who drank at a lower risk level (≤2 standard drinks per day), than in a sample of the general population. Harmful drinking and alcohol dependence were most commonly observed in men, younger age groups, non-commissioned officers and lower ranks as well as reserve and ex-serving groups. Alcohol misuse was clearly associated with poorer general health scores, more role limitations because of physical health problems, and lower social functioning.

**Conclusions:**

Although risky drinking was lower in the military group than in the general population, drinking was associated with poorer health, more limitations because of physical health problems, and poorer social functioning in Defence members. These results highlight the potential benefits for Defence forces in reducing alcohol use among members, in both those groups identified at highest risk, and across the military workforce as a whole.

**Electronic supplementary material:**

The online version of this article (doi:10.1186/s13011-015-0023-4) contains supplementary material, which is available to authorized users.

## Introduction

Historically there has been a strong tradition of alcohol consumption in military populations [1] and moderate consumption is still considered to be an important catalyst for bonding and cohesion in the military [2]. However, there are a number of negative effects of heavy alcohol use. It has been linked to physical conditions, including liver damage, cancers, cardiovascular disease, and injuries [3], and has been shown to be associated with both major depression [4] and increased symptoms of Post-Traumatic Stress Disorder [5]. This study reports measures of alcohol consumption, identifies high risk groups, and examines the association between drinking and general health, limitations because of physical health problems, and social functioning in Australian military personnel.

As well as impacting on health, heavy alcohol use in military settings has been shown to be associated with a number of behavioral and performance issues, such as ‘being passed over for promotion’, arrests for ‘drink driving’ [6] and ‘violence on homecoming’ [7]. Workplace outcomes such as lateness, leaving early, low performance, and injuries are also more common among heavy drinkers in the United States (US) Defense force [8].

The evidence for performance impairment from the effects of a ‘hangover’ in military groups is inconclusive [9]. Nevertheless, some studies have shown that alcohol use disorders are associated with poorer functioning [10–13]. In a number of these studies the poorer functioning was observed in harmful and dependent drinkers, but not in those who drank at lower levels, or in weekly binge drinkers [11, 10]. Therefore, heavy alcohol use, in particular, may have major effects on the health of a Defence force member and their ability to deploy.

Within a military, certain occupational groups or exposures may be associated with alcohol misuse. Higher levels of drinking have been identified among single and younger personnel [1, 11]. Similarly, having problems at home around the time of deployment, and poor unit leadership have been reported as predictors of higher drinking levels [2]. Deploying with one’s parent unit and a high level of camaraderie in the unit have also been associated with higher drinking [2].

Consistent with a perceived culture of drinking in military personnel, studies from the United Kingdom (UK) and the US have shown higher alcohol consumption in the military generally [14], and among Naval personnel [15, 16] compared to civilians. However, results from US studies in a similar period were inconsistent. For example, Polich showed that rates of alcohol abuse were similar between military and civilian groups, once demographic differences had been accounted for [17]. Ballweg reported that while non-drinking was higher in civilians, a higher proportion of military personnel were likely to drink at low risk levels (1-2 standard drinks a day) compared with their civilian counterparts [18].

The first aim of this study was to compare the prevalence of drinking in currently serving and former members of the Australian Defence Force (ADF) with a nationally representative sample of civilians. A 2010 study, limited to currently serving personnel, showed that alcohol use disorders were significantly lower in Australian military personnel compared to the Australian community [19]. To gain a more comprehensive picture the present study also included both current and ex-serving personnel and compared the proportions of abstainers and low risk drinkers in these groups.

The second aim of the study was to identify which groups among current and former ADF personnel were most likely to report harmful drinking and binge drinking. While certain characteristics (such as being young and single), have been previously reported as risk factors of these behaviors [1, 11], the aim was to identify whether other characteristics such as service, rank and employment status (e.g. current or ex-serving) were also risk factors for drinking to a harmful level or binge drinking. The identification of such groups can inform policies to reduce harmful drinking in the ADF.

The final aim was to examine the association between drinking and general health and the ability to function normally. While other studies have shown that those with alcohol dependence have poorer health and work outcomes [11, 13, 12], the present study was able to observe whether those who drink at lower levels also had poorer outcomes.

## Methods

### Study groups

The Bougainville Deployment Health Study and the East Timor Deployment Health Study were cross-sectional surveys, undertaken in 2008 [20, 21], to assess the health and experiences of the current and former ADF members deployed to these countries between 1997 and 2005. All 4,775 veterans known to have deployed to Bougainville were invited to participate. The East Timor study invited a representative sample of 3,999 veterans from a deployment of 19,705. Each study also included comparison groups of personnel in the ADF at the same time but who were not deployed to the country studied (*n* = 2,363 and *n* = 2,501 respectively). For this paper the data from the deployed and comparison groups of these two studies were combined to provide a cohort of serving and former ADF personnel who served in the period from 1997 to 2005, and completed a study survey in 2008. The combined sample invited to take part in the survey was 12,829 (as 809 were in both the Bougainville and East Timor studies).

### Recruitment

An invitation was sent to the 12,829 individuals to complete a survey on paper or online. Reminder cards/emails were sent within one month and follow-up phone calls were then made to non-responders. The period of recruitment for the study was from November 2007 to January 2009. Informed consent for participation in the study was obtained from each participant. Demographic characteristics were available for the full list of people invited to the study from the ADF personnel database (PMKeyS). The overall survey response rates for people who provided complete data on the AUDIT scale for the Bougainville and East Timor studies were 43 % and 41 % respectively (overall 41 %, *N* = 5311). Most people completed the survey online (87 %). The response rates were higher among women, older age groups, Air Force personnel (compared to Navy and Army personnel), officers (compared to lower ranks) and currently serving ADF members (compared to former members, Table 1). In the dataset used for analysis, the proportion of women was 12.9 %.

**Table 1:** Australian Defence Force sample survey response rates by demographic characteristics (numbers and row percentages)

	Responder to AUDIT scale	Non-responder to AUDIT scale/survey	Chi-squared test p-value
Gender			
Male	4628 (41.0)	6671 (59.0)	
Female	683 (44.6)	847 (55.4)	0.0061
Age			
20–29	659 (28.0)	1696 (72.0)	
30–39	2382 (39.8)	3603 (60.2)	
40–49	1629 (48.4)	2024 (51.6)	
50–59	559 (57.3)	417 (42.7)	
60+	81 (56.6)	62 (43.4)	<0.0001
Service			
Navy	1076 (39.1)	1674 (60.9)	
Army	3831 (41.7)	5366 (58.3)	
Air Force	404 (45.8)	478 (54.2)	0.0014
Rank			
Officer	1602 (51.4)	1512 (48.6)	
Non-commissioned Officer	3056 (44.1)	3879 (55.9)	
Other ranks	641 (23.3)	2108 (76.7)	<0.0001
Employment status			
Full time	2883 (50.2)	2864 (49.8)	
Reserve	1723 (45.5)	2062 (54.5)	
Ex-serving	705 (21.4)	2589 (78.6)	<0.0001

These studies were approved by the Australian Defence Human Research Ethics Committee, the Department of Veterans’ Affairs Human Research Ethics Committee, and the University of Queensland Behavioural & Social Sciences Ethical Review Committee.

### National Drug Strategy Household Survey (NDSHS)

Results published from the 2010 National Drug Strategy Household Survey (NDSHS) were used to compare levels of drinking between Australian military personnel and the general population [22]. The NDSHS sample included 26648 people over the age of 12 (94 % over the age of 18). The alcohol consumption patterns compared between the ADF and NDSHS were: abstainers, low risk drinkers (no more than two standard drinks per day), and high risk drinkers (more than two standard drinks per day). In this manuscript we refer to drinking more than two standard drinks per day as ‘risky drinking’.

### Measurements

Alcohol use was compared between the following subgroups of the ADF: age (20-29, 30-39, 40-49 and 50+), gender, service (Navy, Army and Air Force), rank (officer, non-commissioned officer and other ranks), ADF employment status (full-time, reserve or ex-serving) and marital status (single/other, living with partner, married and divorced/separated).

The Alcohol Use Disorders Identification Test (AUDIT) is a 10 item scale which can be used to identify hazardous and harmful patterns of alcohol consumption [23]. The scale focuses on current drinking behaviours and experiences with alcohol in the previous 12 months. The first eight items have five response options that are scored from 0 to 4 and the last two items have three response options scored 0, 2 or 4. The responses are summed to give a score from 0 to 40. People who score 0 are abstainers, while those who score 1-7 are considered low risk drinkers. Scores between 8 and 15 represent people who drink in excess of guidelines for low risk consumption (hazardous drinking; more than 10 grams of alcohol a day) [24]. In the analyses, a score of ≥16 on the AUDIT scale was classified as drinking at a harmful level and a score ≥20 was defined as probable alcohol dependence. Previous studies using the AUDIT scale have reported Cronbach’s alpha reliability coefficients ranging between 0.69 and 0.74, and estimates of the test-retest reliability between 0.81 and 0.98 [25].

The NDSHS report did not include results from the AUDIT scale. However, the first two questions of the AUDIT scale ask about the frequency of drinking and the quantity of alcohol consumed on a typical drinking day. Responses to these questions were used to estimate the number of drinks consumed in a week for each ADF study participant (see Additional file 1: Figure S1). These estimates were compared to data on the level of drinking observed in the general Australian population obtained from the 2010 NDSHS [22].

The third question in the AUDIT scale asks how frequently a respondent has had six or more drinks on one occasion. Responses to this question were used to estimate the prevalence of binge drinking. ‘Binge drinking weekly or more’ and ‘binge drinking monthly or more’ were used as separate binary outcomes in logistic regression models.

Three subscales of the Short Form (36) Health Survey (SF-36) were used to calculate the general health, role physical and social functioning scores of participants. The general health scale has five questions. The role physical scale has four questions about role limitations because of physical health problems. The social functioning scale has two questions about the impact of physical health and emotional problems on normal social activities. Each of these subscales was scored between 0 and 100, with higher scores indicating better health [26]. The SF-36 questions used generally asked about the responders’ experiences in the previous 4 weeks.

### Statistical analysis

The demographic characteristics of responders and non-responders (obtained from the ADF personnel database) were compared using chi-squared tests. The proportions of people drinking at the ‘risky’ and ‘low risk’ levels and the proportions of abstainers were compared between the ADF and NDSHS samples using logistic regression. These comparisons were adjusted for age (10 year groups) and gender to account for the different demographic characteristics of the samples. For the comparisons with NDSHS data, the ADF survey results were not weighted for non-response. However, a sensitivity analysis was also undertaken which weighted the ADF survey data for non-response by service, rank and employment status.

Logistic regression models were used to compare patterns of harmful drinking (AUDIT ≥ 16), alcohol dependence (AUDIT ≥ 20) and binge drinking between subgroups of the ADF sample. Multiple regression models were used to compare general health, role physical and social functioning scores between people in each alcohol consumption category. These comparisons were adjusted for demographic characteristics and smoking status (current smoker, ex-smoker or never smoker), as smoking was hypothesised to be a possible confounding variable [27].

Unless specified otherwise, analyses were weighted for non-response to ensure that results were representative of the Australian Military sample invited to participate. Weights were calculated for strata defined by gender, rank, service and employment status. Statistical analyses were performed using SAS version 9.3 [28] and STATA version 10.1 [29].

## Results

**Fig 1:**
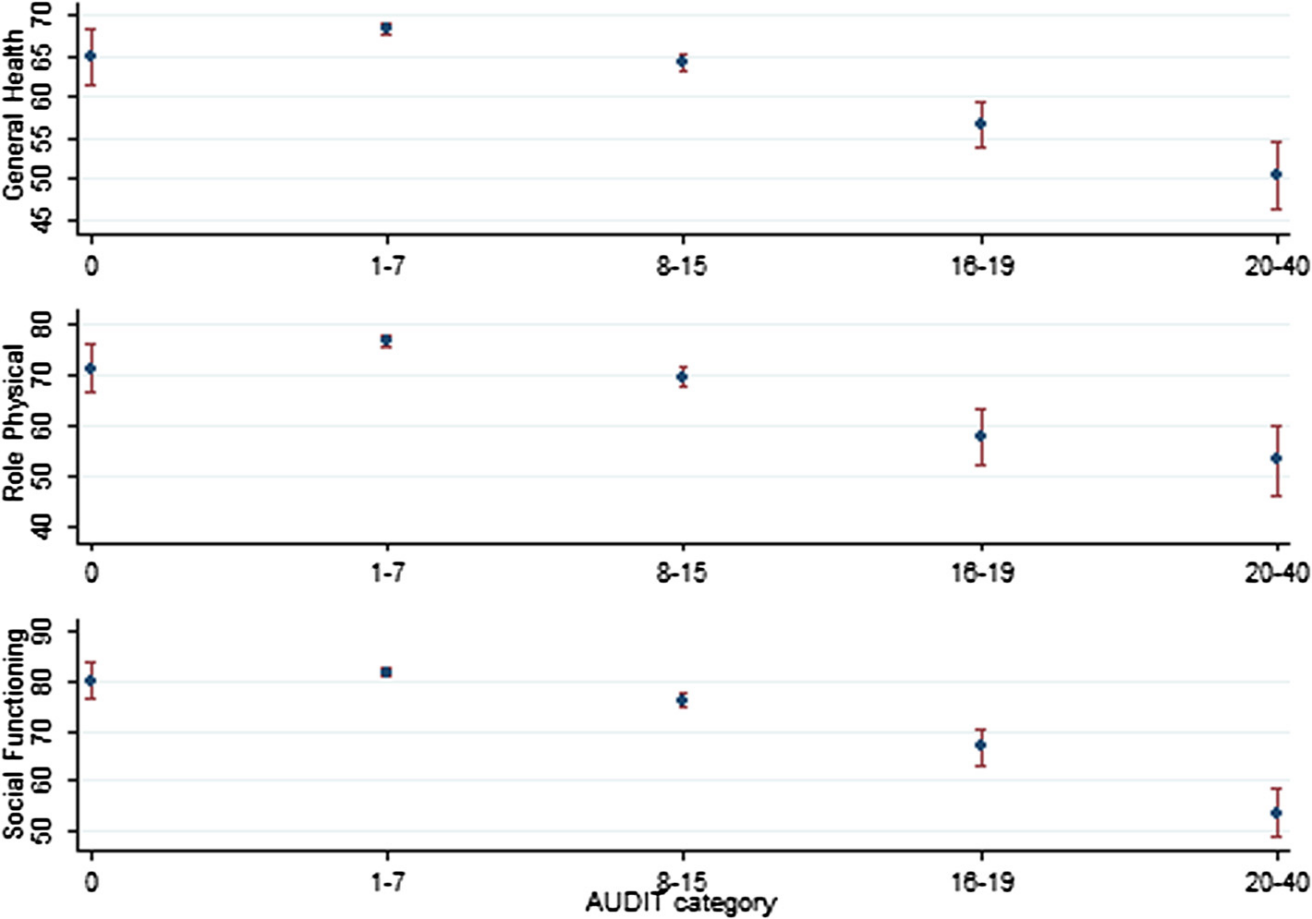
General health, role physical and social functioning, by alcohol use category (AUDIT scale) in the Australian Defence Force sample. Footnote: AUDIT scores 0 = Abstainers, 1-7 = low risk drinkers, 8-15 = hazardous drinking, 16-19 = harmful drink, ≥20 probable alcohol dependence

There were clear and consistent associations between high scores on the AUDIT scale and poorer health outcomes. Mean scores for the general health, role physical (role limitations because of physical health problems) and social functioning scales all decreased with increased AUDIT scores (Fig. 1 and Additional file 2: Table S1). Clear and statistically significant differences were observed for those who drink at a harmful level (AUDIT 16-19) and those who were alcohol dependent (AUDIT ≥20), compared to low risk drinkers (AUDIT 1-7). Those with an AUDIT score of 8-15 also had poorer health scores compared to the low risk drinkers (p-values <0.0001). The health outcomes for low risk drinkers (AUDIT 1-7) were slightly better than for the abstainers (AUDIT = 0), however, these differences were not statistically significant for the general health and social functioning scales. Among those who drank at a low risk level (≤2 drinks a day), 27 % reported ‘binge drinking’ at least monthly. However, these ‘binge drinkers’ did not have significantly poorer scores on the general health and role physical and social functioning subscales compared to low risk drinkers who did not report ‘binge drinking’ (Additional file 3: Table S2).

Compared to the NDSHS sample, the current and former ADF members were less likely to be abstainers (OR 0.28 95 % CI (0.24, 0.33)), and less likely to drink at a risky level (on average, more than 2 drinks a day) (OR 0.59 (95 % CI (0.54, 0.64)), but more likely to drink at a low risk level (OR 2.30 95 % CI (2.12, 2.47)) (Table 2). These patterns were observed in both men and women. The results changed only marginally when the ADF data were weighted for non-response (Additional file 4: Table S3).

**Table 2:** Drinking behaviours in Australian Defence Force (ADF) sample compared to the general population sample (NDSHS)

	Percentage of risky drinkers in ADF sample (%)^a^	Percentage of risky drinkers in civilian sample (%)	Percentage of abstainers in ADF sample (%)	Percentage of abstainers in civilian sample (%)	Percentage of low risk drinkers in ADF sample (%)^b^	Percentage of low risk drinkers in civilian sample (%)
Males						
20–29	109/551 (19.8 %)	36.1 %	20/551 (3.6 %)	13.9 %	422/551 (76.6 %)	50.0 %
30–39	398/2010 (19.8 %)	31.1 %	55/2010 (2.7 %)	13.5 %	1557/2010 (77.5 %)	55.4 %
40–49	333/1475 (22.6 %)	30.8 %	57/1475 (3.9 %)	12.5 %	1085/1475 (73.6 %)	56.7 %
50–59	136/530 (25.7 %)	30.8 %	32/530 (6.0 %)	12.8 %	362/530 (68.3 %)	56.4 %
60+	22/81 (27.2 %)	27.9 %	1/81 (1.2 %)	13.5 %	58/81 (71.6 %)	58.6 %
Females						
20–29	12/113 (10.6 %)	17.4 %	4/113 (3.5 %)	15.6 %	97/113 (85.8 %)	67.0 %
30–39	25/382 (6.5 %)	11.3 %	20/382 (5.2 %)	17.9 %	337/382 (88.2 %)	70.8 %
40–49	19/151 (12.6 %)	12.8 %	17/151 (11.3 %)	16.0 %	115/151 (76.2 %)	71.2 %
50–59	4/28 (14.3 %)	11.9 %	3/28 (10.7 %)	20.2 %	21/28 (75.0 %)	68.0 %
60+	0/1 (0 %)	7.5 %	0/1 (0 %)	26.0 %	1/1 (100 %)	66.5 %
	Risky Drinkers	95 % CI	Abstainers	95 % CI	Low risk Drinkers	95 % CI
	Odds ratio		Odds ratio		Odds ratio	
Males	0.64	(0.60, 0.69)	0.25	(0.21, 0.29)	2.25	(2.11, 2.39)
Females	0.67	(0.51, 0.85)	0.34	(0.25, 0.46)	2.32	(1.87, 2.88)
Overall	0.59	(0.54, 0.64)	0.28	(0.24, 0.33)	2.30	(2.12, 2.47)

^a^Risky drinkers were those who drank more than 2 standard drinks in a day

^b^No more than 2 standard drinks in a day

AUDIT scores 0 = Abstainers, 1-7 = low risk drinkers, 8-15 = hazardous drinking, 16-19 = harmful drink, ≥20 probable alcohol dependence

The median AUDIT score in the ADF sample was 5.0 (interquartile range 3-9) and the mean was 6.9 (standard deviation 5.3). Overall 4 % of participants were abstainers and 60 % were low risk drinkers. Hazardous drinking (AUDIT ≥ 8) was reported by 36 % of responders, whereas the percentages of those drinking at a harmful level (AUDIT ≥ 16) and those with probable alcohol dependence (AUDIT ≥ 20) were 9 % and 4 % respectively.

Men were more likely than women to drink at a harmful level (AUDIT ≥ 16), as were those aged 20-29 compared to older age groups (Table 3). The proportion of responders drinking at a harmful level was similar across the older age groups (above the age of 30). Harmful drinking was most commonly reported among those with ranks below the officer level, reserves and ex-serving members. People who were married were less likely to report a harmful level of drinking. Harmful drinking was lower among Air Force personnel, however these differences were not statistically significant. Similar patterns were observed with alcohol dependence (AUDIT ≥ 20), however, age group and lower rank were no longer statistically significantly associated with this behaviour. Binge drinking was also most common in men, those aged 20-29, Army, ex-serving members, and non-commissioned officers and lower ranks. Binge drinking was least common among Air Force and married responders (Table 4).

**Table 3:** Drinking patterns by demographic characteristics in the Australian Defence Force sample – frequencies, weighted percentages, odds ratios and 95 % confidence intervals

	AUDIT 0	AUDIT 1-7	AUDIT 8-15	AUDIT 16-19	AUDIT 20-40	AUDIT ≥ 16	P-value	AUDIT ≥ 20	P-value
						Odds ratio 95 % CI^a^		Odds ratio 95 % CI^a^	
Gender									
Male	164 (3.6)	2798 (58.3)	1279 (28.4)	202 (5.1)	173 (4.6)	1 (Reference)		1 (Reference)	
Female	44 (7.5)	509 (73.1)	102 (14.7)	14 (2.2)	14 (2.5)	0.44 (0.30, 0.64)	<0.0001	0.54 (0.32, 0.92)	0.02
Age									
20-29	24 (3.5)	343 (49.5)	224 (34.4)	40 (7.1)	27 (5.5)	1.39 (1.01, 1.93)	0.05	1.32 (0.80, 2.19)	0.28
30-39	75 (3.5)	1499 (60.4)	637 (27.4)	94 (4.7)	75 (3.9)	1 (Reference)		1 (Reference)	
40-49	74 (4.7)	1049 (63.7)	384 (23.6)	54 (3.4)	63 (4.6)	1.00 (0.79, 1.25)	0.96	1.20 (0.86, 1.67)	0.29
50+	35 (5.3)	416 (64.1)	136 (21.4)	28 (4.8)	22 (4.4)	1.14 (0.84, 1.55)	0.40	1.08 (0.69, 1.68)	0.74
Marital Status									
Single/Other	26 (4.6)	310 (50.2)	190 (32.8)	33 (6.7)	25 (5.8)	1.68 (1.22, 2.30)	0.001	1.51 (0.94, 2.41)	0.09
Living with partner	24 (3.6)	372 (53.7)	201 (31.8)	34 (5.8)	28 (5.2)	1.52 (1.12, 2.05)	0.007	1.41 (0.92, 2.16)	0.11
Married	131 (4.1)	2210 (64.6)	790 (24.0)	105 (3.5)	100 (3.7)	1 (Reference)		1 (Reference)	
Separated/Divorced	21 (4.8)	260 (53.6)	131 (28.4)	27 (7.0)	25 (6.2)	1.95 (1.44, 2.65)	<0.0001	1.66 (1.07, 2.56)	0.02
Service									
Navy	30 (3.2)	654 (59.4)	309 (28.8)	47 (5.2)	35 (3.4)	0.95 (0.75, 1.22)	0.71	0.76 (0.54, 1.07)	0.12
Army	155 (4.1)	2369 (59.8)	993 (26.6)	157 (4.6)	147 (4.9)	1 (Reference)		1 (Reference)	
Air Force	23 (5.9)	284 (73.9)	79 (20.1)	12 (3.9)	5 (2.1)	0.77 (0.52, 1.15)	0.20	0.45 (0.16, 1.27)	0.13
Rank									
Officer	50 (3.3)	1138 (70.3)	342 (21.4)	42 (2.6)	30 (2.4)	0.69 (0.49, 0.97)	0.03	0.76 (0.45, 1.29)	0.31
Non-commissioned Officer	130 (4.4)	1816 (58.3)	848 (27.8)	135 (4.8)	127 (4.6)	1.16 (0.87, 1.55)	0.31	1.24 (0.81, 1.89)	0.31
Other ranks	28 (4.0)	353 (53.7)	191 (29.5)	39 (6.7)	30 (6.0)	1 (Reference)		1 (Reference)	
Employment status									
Full time	105 (3.7)	1859 (64.0)	754 (26.5)	96 (3.5)	66 (2.4)	1 (Reference)		1 (Reference)	
Reserve	62 (3.6)	1091 (62.8)	438 (26.2)	65 (3.9)	60 (3.6)	1.37 (1.12, 1.67)	0.003	1.57 (1.18, 2.10)	0.0023
Ex-serving	41 (5.3)	357 (50.9)	189 (27.3)	55 (7.8)	61 (8.7)	3.09 (2.43, 3.93)	<0.0001	3.91 (2.82, 5.43)	<0.0001

NB. AUDIT score ≥16 also includes those with an AUDIT score ≥20

^a^ Odds ratios compare drinking behaviours between the categories of the demographic characteristics

**Table 4:** Binge drinking (6 or more drinks on one occasion) in the Australian Defence Force sample by demographic characteristics – frequencies, weighted percentages, odds ratios and 95 % confidence intervals

	Never	Less than monthly	Monthly	Weekly	Daily or almost daily	Weekly or daily binge drinking	p-value	Monthly or more binge drinking	p-value
						Odds ratio 95 % CI^a^		Odds ratio 95 % CI^a^	
Gender									
Male	868 (18.1)	1931 (39.7)	892 (19.1)	830 (18.5)	182 (4.6)	1 (Reference)		1 (Reference)	
Female	306 (43.2)	257 (38.7)	67 (9.3)	55 (8.0)	4 (0.8)	0.30 (0.23, 0.39)	<0.0001	0.27 (0.22, 0.32)	<0.0001
Age									
20–29	84 (12.3)	255 (35.4)	184 (27.5)	141 (22.4)	13 (2.4)	1.21 (0.96, 1.51)	0.10	1.65 (1.38, 1.97)	<0.0001
30–39	455 (18.7)	1080 (43.7)	446 (18.3)	374 (16.0)	61 (3.3)	1 (Reference)		1 (Reference)	
40–49	408 (24.7)	658 (39.2)	235 (13.7)	274 (17.0)	77 (5.3)	1.20 (1.04, 1.39)	0.01	0.91 (0.81, 1.03)	0.15
50+	227 (34.6)	195 (29.2)	94 (14.3)	96 (15.3)	35 (6.5)	1.25 (1.02, 1.52)	0.03	1.00 (0.85, 1.19)	0.98
Marital Status									
Single/Other	83 (13.0)	222 (39.3)	119 (20.4)	129 (23.8)	15 (3.5)	1.66 (1.33, 2.05)	<0.0001	1.50 (1.25, 1.81)	<0.0001
Living with partner	96 (14.1)	263 (38.9)	146 (24.1)	124 (20.0)	18 (3.0)	1.28 (1.05, 1.56)	0.01	1.47 (1.25, 1.72)	<0.0001
Married	667 (20.4)	1410 (42.6)	557 (17.0)	492 (15.6)	115 (4.4)	1 (Reference)		1 (Reference)	
Separated/Divorced	78 (16.1)	168 (36.6)	87 (20.2)	87 (19.5)	28 (7.5)	1.43 (1.16, 1.76)	0.0007	1.52 (1.28, 1.82)	<0.0001
Service									
Navy	192 (17.4)	480 (43.5)	210 (19.5)	176 (16.5)	31 (3.0)	0.84 (0.72, 0.98)	0.03	0.99 (0.87, 1.13)	0.91
Army	867 (21.8)	1529 (38.3)	683 (17.4)	667 (17.9)	147 (4.6)	1 (Reference)		1 (Reference)	
Air Force	115 (28.2)	178 (41.4)	66 (17.1)	42 (10.5)	8 (2.8)	0.58 (0.44, 0.77)	0.0002	0.77 (0.64, 0.92)	0.004
Rank									
Officer	460 (28.7)	707 (43.0)	241 (14.6)	179 (11.3)	33 (2.4)	0.74 (0.58, 0.94)	0.01	0.73 (0.61, 0.89)	0.0013
Non-commissioned Officer	595 (19.5)	1230 (38.9)	574 (18.0)	582 (19.0)	128 (4.6)	1.31 (1.06, 1.62)	0.01	1.18 (0.99, 1.41)	0.06
Other ranks	117 (17.4)	247 (37.6)	139 (21.0)	123 (19.0)	25 (4.9)	1 (Reference)		1 (Reference)	
Employment status									
Full time	573 (19.1)	1242 (42.2)	572 (19.8)	474 (16.6)	67 (2.3)	1 (Reference)		1 (Reference)	
Reserve	437 (24.5)	708 (40.4)	274 (15.9)	268 (15.7)	60 (3.5)	1.09 (0.96, 1.23)	0.20	0.97 (0.87, 1.07)	0.51
Ex-serving	164 (21.5)	238 (34.1)	113 (16.7)	143 (19.7)	59 (8.0)	1.67 (1.39, 2.00)	<0.0001	1.27 (1.09, 1.49)	0.003

NB Monthly or more binge drinking also includes those who binge drink weekly or daily

^a^Odds ratios compare drinking behaviours between the categories of the demographic characteristics

## Discussion

In the results presented, alcohol misuse is clearly associated with poorer general health, increased difficulties and limitations with work and daily activities, and reduced social functioning in current and former members of the ADF. This suggests that alcohol consumption among Australian military personnel has the potential to limit Defence capacity. As such, strategies to reduce drinking within the ADF may be particularly beneficial in maintaining a healthy and productive workforce. While other studies have shown that alcohol misuse is associated with a lower level of functioning [10–13], the effects were typically observed in those who drink at a harmful level or are alcohol dependent.

Although low risk drinkers had slightly better health scores than abstainers, there was a gradual and statistically significant decline in general health and social functioning, and more role limitations because of physical health problems, with increased alcohol misuse. The abstainers may contain a number of former drinkers who no longer drink because of previous problems with alcohol. However, it has also been hypothesised that abstainers may have poorer social relationships than light or moderate drinkers, which may contribute to poorer health in this group [30].

While we controlled for demographic characteristics and smoking status, it is unclear to what extent alcohol use was a primary contributor to reduced health and functioning, or whether the responders may have been using alcohol to cope with other physical or mental health conditions which were also associated with poorer functioning. Longitudinal studies of alcohol use may help researchers to more clearly determine the order of these events.

Consistent with a number of other studies, married personnel were less likely than single persons to report harmful drinking and binge drinking [1, 31, 32], perhaps due to different social and recreational activities undertaken by married people [33]. Also consistent with previous research harmful drinking was most commonly observed among men [6, 34, 31, 32] and younger age groups (20-29 years) [14, 31]. In addition, we found that non-commissioned officers, lower ranks and reserve and ex-serving personnel were more likely to report harmful drinking. These subgroups were also most likely to report ‘binge drinking’ at least monthly.

It is unclear whether increased alcohol consumption among ex-serving members pre-dated (and perhaps contributed to) their departure from the Australian Military or if their alcohol use increased after discharge. Despite assurances of confidentiality, it is possible that members who left the Australian Military may have been more comfortable disclosing their alcohol consumption than serving members.

Overall Navy and Army personnel were more likely to report binge drinking at least monthly than Air Force personnel. Differences in alcohol use between the services may be due to differences in entry requirements and the occupational roles of each service, or to different drinking cultures in each group. Likewise differences in the nature of operational deployments between the services may also impact on drinking behaviours [35]. These results are consistent with other studies that have shown fewer alcohol problems among Air Force personnel [14, 32].

The subgroups identified as having more alcohol problems and who were more likely to binge drink, may benefit from specific interventions aimed to reduce supply and consumption of alcohol. Recent studies from Australia and the UK have shown that while overall alcohol consumption in the population has fallen in the past decade, alcohol related harm has continued to rise [36–38]. Therefore, it has been argued that as well as aiming to reduce overall consumption, specific and targeted interventions are required to change the behaviour of the ‘hardened drinkers’ [37].

There were fewer current and former ADF members who drank more than 2 standard drinks a day than in the general Australian population. There were also fewer abstainers among current and former Australian Military members, but more people who drank at a lower risk level, than in the general Australian population. It has been suggested that a large proportion of the costs to the ADF associated with drinking may be attributable to ‘low risk drinkers’ who occasionally drink heavily [39]. In our analysis, more than 25 % of the low risk drinkers (who averaged less than 2 standard drinks per day) reported ‘binge drinking’ at least monthly. However, this group, were not shown to have worse health and functioning outcomes, compared to ‘low risk riskers’ who did not ‘binge drink’. A culture of social drinking and events in the ADF may explain why there were fewer abstainers. In contrast the finding of fewer risky drinkers in the ADF group may be due in part to a ‘healthy soldier effect,’ stemming from a requirement to maintain a good standard of fitness while serving [40].

The study reported a slightly higher percentage of serving members drinking above the harmful level (5.9 %, AUDIT ≥16) than a 2010 of serving ADF members (3.7 %, AUDIT ≥16) [19]. Comparing our findings with the international literature, we found that the percentage of males with a high level of alcohol problems was lower than observed in a UK study of military personnel conducted in 2003 (16 %, AUDIT ≥16) [11], but the percentages drinking more than 2 standard drinks per day were higher than reported in studies of US military personnel [41, 42]. In a comparison of drinking behaviours between US and UK military personnel, Sundin et al highlighted that the command-directed alcohol treatment program and a military wide campaign to reduce alcohol consumption, may have contributed to reduced alcohol misuse in the US military, relative to other countries [43].

The result that fewer ADF members drank more than 2 standard drinks a day than in a sample of Australian civilians, contrasts with some US and UK studies that have reported more heavy alcohol users in military personnel compared to civilians [42, 14]. These results are inconsistent with other US and UK studies which found fewer differences in drinking patterns between veterans and those without military experience [41, 44]. Different outcome measures used to measure alcohol misuse in each study, as well as different levels of drinking in the population of each country [45], may explain some of the differences in findings. Although the prevalence of drinking and alcohol misuse varies between UK, US and Australian studies, research has indicated that a there is a perceived culture of drinking within the Defence Forces of each of these countries [43, 46, 14, 41]. Therefore the findings presented from the ADF may also be useful to inform strategies in other militaries.

Changing drinking behaviours within military groups is likely to be challenging given established behaviours and practices developed over a number of years. The suggestion that drinking is seen to be an important catalyst for cohesion in military groups [2] is another potential barrier in reducing alcohol use. Nevertheless, since these data were collected (2008-2009), the ADF has commissioned an independent report on ‘Use of Alcohol in the Australian Defence Force’ [46] and developed an Alcohol Management Strategy. The report made a series of recommendations, encouraged a proactive (as opposed to reactive) approach, and endorsed an overall preventative stance with regard to alcohol use. One notable policy recommendation from the independent report was to change the focus of alcohol use in the ADF, so that instead of decisions being made about the situations where alcohol should be banned, decisions should be based on the question ‘in which situations should alcohol be permitted?’ [46].

In 2013 the ADF also produced a “Leaders guide alcohol management” document, which highlighted the role of ADF supervisors at all levels in addressing alcohol related issues and how more senior members should lead by example by upholding particular standards of behaviour [47]. This guide is important, especially because our results indicated that non-commissioned officers had higher levels of binge drinking compared to other ranks.

As the data we presented pre-date a number of these initiatives, our results provide baseline data against which these preventative initiatives can be evaluated. Further follow-up of ADF cohorts would help detect whether there are meaningful changes in patterns of alcohol use of personnel and determine the effectiveness of any new policies introduced to reduce alcohol related harm.

The survey response rates were between 41 % and 43 %, so there may be some response bias. In the analysis we have reported, where possible, results for non-response using weights defined by service, rank and employment status. The measures on alcohol use and health used were self-reported, as opposed to more objective measures. Although the survey was confidential, the levels of alcohol use presented may be underestimates if serving members underreported current alcohol use. However, previous studies have shown the AUDIT scale to be a valid measure [48].

## Conclusion

Although the proportion of risky drinkers among current and former ADF members was shown to be lower than that observed in the general Australian population, reform of drinking practices within the ADF is likely to improve both health and performance. As well as focusing on broad strategies aimed at reducing drinking across their whole workforce, militaries may also benefit from strategies focused on those groups at highest risk of alcohol misuse and binge drinking.

## Additional files

Additional file 1: Figure S1. Classification of weekly alcohol consumptions based on responses to the first two AUDIT questions. (DOCX 14 kb) Additional file 2: Table S1. General health, role physical and social functioning, by alcohol use category (AUDIT scale) in the Australian Defence Force sample (*n* = 4503). (DOCX 15 kb) Additional file 3: Table S2. General health, role physical and social functioning for binge drinkers and non-binge drinkers in the Australian Defence Force sample. (DOCX 16 kb) Additional file 4: Table S3. Drinking behaviours in Australian Defence Force sample compared to the civilian sample (NDSHS) – ADF sample weighted for non-response. (DOCX 16 kb)
