# Supplementary material for: Alcohol use in the military: associations with health and wellbeing
Source: Subst Abuse Treat Prev Policy. 2015 Jul 28;10:27. doi: 10.1186/s13011-015-0023-4 (PMC4518507; doi:10.1186/s13011-015-0023-4)
Supplement: Additional file 1: Figure S1. — Classification of weekly alcohol consumptions based on responses to the first two AUDIT questions. (DOCX 14 kb) [file 13011_2015_23_MOESM1_ESM.docx]

Supplementary Figure 1: Classification of weekly alcohol consumptions based on responses to the first two AUDIT questions.

|  |  | Q2: How many standard drinks do you have on a typical day when you are drinking? | | | | | |
| --- | --- | --- | --- | --- | --- | --- | --- |
|  |  | None | 1 or 2 | 3 or 4 | 5 or 6 | 7 to 9 | 10 + |
| Q1: How often do you have a drink containing alcohol? | Never |  |  |  |  |  |  |
|  | Monthly or less |  |  |  |  |  |  |
|  | 2 to 4 times a month |  |  |  |  |  |  |
|  | 2 to 3 times a week |  |  |  |  |  |  |
|  | 4 or more times a week |  |  |  |  |  |  |

The following classifications were used based on the midpoints of each category:

Green section = abstainers

Yellow section = low risk drinkers (≤2 drinks per day)

Red section = risky drinkers (>2 drinks per day)
