# Supplementary material for: Alcohol use in the military: associations with health and wellbeing
Source: Subst Abuse Treat Prev Policy. 2015 Jul 28;10:27. doi: 10.1186/s13011-015-0023-4 (PMC4518507; doi:10.1186/s13011-015-0023-4)
Supplement: Additional file 2: Table S1. — General health, role physical and social functioning, by alcohol use category (AUDIT scale) in the Australian Defence Force sample (n = 4503). (DOCX 15 kb) [file 13011_2015_23_MOESM2_ESM.docx]

Supplementary Table 1: General health, role physical and social functioning, by alcohol use category (AUDIT scale) in the Australian Defence Force sample (n=4503)

| AUDIT score | 0 | 1-7 | 8-15 | 16-19 | 20-40 |
| --- | --- | --- | --- | --- | --- |
| General Health |  |  |  |  |  |
| Score (95% CI) | 64.9 (61.4, 68.5) | 68.3 (67.7, 69.0) | 64.1 (63.0, 65.2) | 56.5 (53.8, 59.3) | 50.4 (46.3, 54.5) |
| Model estimate (95% CI) | 0 (Reference) | 3.4 (-0.3, 7.0) | 0.8 (-2.9, 4.6) | -8.4 (-12.9, -3.9) | -14.6 (-20.0, -9.1) |
| p-value |  | 0.07 | 0.67 | 0.0002 | <0.0001 |
|  |  |  |  |  |  |
| Role physical |  |  |  |  |  |
| Score (95% CI) | 71.1 (66.4, 75.9) | 76.8 (75.7, 77.9) | 69.5 (67.5, 71.5) | 57.9 (52.5, 63.3) | 53.1 (46.1, 60.0) |
| Model estimate (95% CI) | 0 (Reference) | 5.7 (0.8, 10.6) | -1.7 (-6.9, 3.6) | -13.3 (-20.5, -6.0) | -18.1 (-26.6, -9.6) |
| p-value |  | 0.02 | 0.54 | 0.0003 | <0.0001 |
|  | | | | | |
| Social functioning |  |  |  |  |  |
| Score (95% CI) | 80.3 (76.8, 83.8) | 81.9 (81.2, 82.7) | 76.3 (75.0, 77.7) | 66.7 (62.8, 70.6) | 53.6 (48.9, 58.3) |
| Model estimate (95% CI) | 0 (Reference) | 1.7 (-2.0, 5.3) | -4.0 (-7.8, -0.2) | -13.6 (-18.8, -8.3) | -26.7 (-32.6, -20.8) |
| p-value |  | 0.37 | 0.04 | <0.0001 | <0.0001 |
|  |  |  |  |  |  |

Adjusted for age (20-29, 30-39, 40-49 and 50+), sex, service (Navy, Army and RAAF), rank (officer, non-commissioned officer and other ranks), Employment status (regular, reserve or ex-serving) and smoking status (current, former, or never smoker).
