# Supplementary material for: Alcohol use in the military: associations with health and wellbeing
Source: Subst Abuse Treat Prev Policy. 2015 Jul 28;10:27. doi: 10.1186/s13011-015-0023-4 (PMC4518507; doi:10.1186/s13011-015-0023-4)
Supplement: Additional file 3: Table S2. — General health, role physical and social functioning for binge drinkers and non-binge drinkers in the Australian Defence Force sample. (DOCX 16 kb) [file 13011_2015_23_MOESM3_ESM.docx]

Supplementary Table 2: General health, role physical and social functioning for binge drinkers and non-binge drinkers in the Australian Defence Force sample

|  | | Low risk drinkers ^A^ (n=3199) | | | | Overall (n=4580) | | | |
| --- | --- | --- | --- | --- | --- | --- | --- | --- | --- |
|  | | Binge drinker ^B^ | | Non-binge drinker | | Binge drinker ^B^ | | Non-binge drinker | |
| General Health | |  | |  | |  | |  | |
| Score (95% CI) | | 67.1 (65.8, 68.5) | | 67.3 (66.6, 68.1) | | 64.0 (63.1, 65.0) | | 67.1 (66.4, 67.8) | |
|  | |  | |  | |  | |  | |
| Model estimate (95% CI) | | -0.2 (-1.9, 1.5) | | 0 (Reference) | | -3.1 (-4.4, -1.8) | | 0 (Reference) | |
| p-value | | 0.82 | | - | | <0.0001 | | - | |
|  | |  | |  | |  | |  | |
| Role physical | |  | |  | |  | |  | |
| Score (95% CI) | | 73.9 (71.4, 76.3) | | 75.5 (74.2, 76.7) | | 70.2 (68.5, 71.8) | | 74.9 (73.8, 76.0) | |
|  | |  | |  | |  | |  | |
| Model estimate (95% CI) | | -1.6 (-4.5, 1.3) | | 0 (Reference) | | -4.7 (-6.9, 2.5) | | 0 (Reference) | |
| p-value | | 0.28 | | - | | <0.0001 | | - | |
|  | | | | | | | | | |
| Social functioning |  | |  | |  | |  | |  |
| Score (95% CI) | | 79.2 (77.6, 80.9) | | 80.6 (79.7, 81.5) | | 75.8 (74.7, 77.0) | | 80.5 (79.8, 81.3) | |
| Model estimate (95% CI) | | -1.4 (-3.4, -0.7) | | 0 (Reference) | | -4.7 (-6.3, -3.1) | | 0 (Reference) | |
|  | |  | |  | |  | |  | |
| p-value | | 0.19 | |  | | <0.0001 | | - | |
|  | |  | |  | |  | |  | |

Adjusted for age (20-29, 30-39, 40-49 and 50+), sex, service (Navy, Army and RAAF), rank (officer, non-commissioned officer and other ranks), Employment status (regular, reserve or ex-serving) and smoking status (current, former, or never smoker).

^A^ On average no more than 2 standard drinks per day

^B^ 6 or more drinks on one occasion, monthly or more
