# Supplementary material for: Alcohol use in the military: associations with health and wellbeing
Source: Subst Abuse Treat Prev Policy. 2015 Jul 28;10:27. doi: 10.1186/s13011-015-0023-4 (PMC4518507; doi:10.1186/s13011-015-0023-4)
Supplement: Additional file 4: Table S3. — Drinking behaviours in Australian Defence Force sample compared to the civilian sample (NDSHS) – ADF sample weighted for non-response. (DOCX 16 kb) [file 13011_2015_23_MOESM4_ESM.docx]

**Supplementary Table 3**: Drinking behaviours in Australian Defence Force sample compared to the civilian sample (NDSHS) – ADF sample weighted for non-response.

|  | Percentage of risky drinkers in ADF sample (%)^A^ | Percentage of risky drinkers in civilian sample (%) | Percentage of abstainers in ADF sample (%) | Percentage of abstainers in civilian sample (%) | Percentage of low risk drinkers in ADF sample (%)^B^ | Percentage of low risk drinkers in civilian sample (%) |
| --- | --- | --- | --- | --- | --- | --- |
| Males |  |  |  |  |  |  |
| 20-29 | 350/1548 (22.6%) | 36.1% | 52/1548 (3.4%) | 13.9% | 1146/1548 (74.0%) | 50.0% |
| 30-39 | 1027/4967 (20.7%) | 31.1% | 143/4967 (2.9%) | 13.5% | 3797/4967 (76.4%) | 55.4% |
| 40-49 | 771/3314 (23.3%) | 30.8% | 131/3314 (4.0%) | 12.5% | 2412/3314 (72.8%) | 56.7% |
| 50-59 | 311/1167 (26.6%) | 30.8% | 68/1167 (5.8%) | 12.8% | 788/1167 (67.5%) | 56.4% |
| 60+ | 56/220 (25.5%) | 27.9% | 3/220 (1.4%) | 13.5% | 161/220 (73.2%) | 58.6% |
|  |  |  |  |  |  |  |
| Females |  |  |  |  |  |  |
| 20-29 | 27/269 (10.0%) | 17.4% | 11/269 (4.1%) | 15.6% | 231/269 (85.9%) | 67.0% |
| 30-39 | 67/956 (7.0%) | 11.3% | 65/956 (6.8%) | 17.9% | 824/956 (86.2%) | 70.8% |
| 40-49 | 43/339 (12.7%) | 12.8% | 42/339 (12.4%) | 16.0% | 254/339 (74.9%) | 71.2% |
| 50-59 | 10/63 (15.8%) | 11.9% | 6/63 (9.5%) | 20.2% | 47/63 (74.6%) | 68.0% |
| 60+ | 1/2 (50.0%) | 7.5% | 0/2 (0%) | 26.0% | 1/2 (50.0%) | 66.5% |
|  |  |  |  |  |  |  |
|  | Risky Drinkers  Odds ratio | 95%CI | Abstainers Odds ratio | 95%CI | Low risk Drinkers  Odds ratio | 95%CI |
| Males | 0.64 | (0.60, 0.69) | 0.25 | (0.22, 0.28) | 2.25 | (2.11, 2.39) |
| Females | 0.71 | (0.59, 0.85) | 0.40 | (0.33, 0.49) | 2.09 | (1.82, 2.41) |
| Overall | 0.63 | (0.59, 0.67) | 0.30 | (0.27, 0.33) | 2.16 | (2.04, 2.28) |

^A^ Risky drinkers were those who drank more than 2 standard drinks in a day

^B^ No more than 2 standard drinks in a day

NB. ADF sample results weighted by Service (Navy, Army and RAAF), rank (officer, non-commissioned officer and other ranks) and employment status (full-time, reserve and ex-serving)
